# Development of a Recombinant RBD Subunit Vaccine for SARS-CoV-2

**DOI:** 10.3390/v13101936

**Published:** 2021-09-26

**Authors:** Yi-Sheng Sun, Jing-Jing Zhou, Han-Ping Zhu, Fang Xu, Wen-Bin Zhao, Hang-Jing Lu, Zhen Wang, Shu-Qing Chen, Ping-Ping Yao, Jian-Min Jiang, Zhan Zhou

**Affiliations:** 1 Key Lab of Vaccine, Prevention and Control of Infectious Disease of Zhejiang Province, Zhejiang Provincial Center for Disease Control and Prevention, Hangzhou 310015, China; yishengfirst@zju.edu.cn (Y.-S.S.); hpzhu@cdc.zj.cn (H.-P.Z.); fxu@cdc.zj.cn (F.X.); hjlu@cdc.zj.cn (H.-J.L.); wangzhen@cdc.zj.cn (Z.W.); 2 Zhejiang Provincial Key Laboratory of Anti-Cancer Drug Research, Institute of Drug Metabolism and Pharmaceutical Analysis, College of Pharmaceutical Sciences, Zhejiang University, Hangzhou 310058, China; 21819048@zju.edu.cn (J.-J.Z.); pharmacy_zwb@zju.edu.cn (W.-B.Z.); chenshuqing@zju.edu.cn (S.-Q.C.); 3 Innovation Institute for Artificial Intelligence in Medicine, Zhejiang University, Hangzhou 310018, China; 4 Alibaba-Zhejiang University Joint Research Center of Future Digital Healthcare, Hangzhou 311121, China

**Keywords:** COVID-19, RBD-Fc, fusion protein, vaccine, neutralizing antibody, cellular immune response

## Abstract

The novel coronavirus pneumonia (COVID-19) pandemic is a great threat to human society and now is still spreading. Although several vaccines have been authorized for emergency use, only one recombinant subunit vaccine has been permitted for widespread use. More subunit vaccines for COVID-19 should be developed in the future. The receptor binding domain (RBD), located at the S protein of SARS-CoV-2, contains most of the neutralizing epitopes. However, the immunogenicity of RBD monomers is not strong enough. In this study, we fused the RBD-monomer with a modified Fc fragment of human IgG1 to form an RBD-Fc fusion protein. The recombinant vaccine candidate based on the RBD-Fc protein could induce high levels of IgG and neutralizing antibody in mice, and these could last for at least three months. The secretion of IFN-γ, IL-2 and IL-10 in the RBD-stimulated splenocytes of immunized mice also increased significantly. Our results first showed that the RBD-Fc vaccine could induce both humoral and cellular immune responses and might be an optional strategy to control COVID-19.

## 1. Introduction

The novel coronavirus pneumonia (COVID-19) that broke out in Wuhan, Hubei Province, in December 2019, was induced by the SARS-CoV-2 virus [1]. To date, COVID-19 has infected more than 200 million people across countries and regions, causing more than 4 million deaths [2]. Currently, considerable strategies targeting SARS-CoV-2 are being developed, such as using casirivimab and imdevimab as an antibody cocktail therapy [3]. Several repurposed drugs such as remdesivir and molnupiravir are also being tested in clinical trials [4]. However, there are no specific antiviral agents curing the illness, and the number of infection cases has been dramatically increasing. Hence, it is an urgent task for us to control the spread of the pandemic and ease the severe burden on human society.

Vaccines are an effective prophylactic measure for controlling the epidemic. Luckily, in spite of several limitations, there are four types of SARS-CoV-2 vaccines authorized for emergency use: the inactivated SARS-CoV-2 vaccines such as BBIBP-CorV, the recombinant adenovirus-vectored COVID-19 vaccines such as Ad5-vectored COVID-19 vaccine and ChAdOx1, and the mRNA vaccines such as mRNA-1273 and BNT162b2 [5,6,7,8]. As for the recombinant subunit vaccine, only one product (ZF2001) based on the tandem-repeat dimeric RBD protein has been permitted for widespread use [9]. Recombinant subunit vaccine, such as the successful hepatitis B vaccine, is convenient for large-scale production and transportation. What is more, the recombinant subunit vaccines, such as the RBD vaccine, are protein antigens that are much safer than the nucleic acid vaccines. Development of more subunit vaccines for SARS-CoV-2 might be an alternative potent approach for defeating COVID-19. Recently, several studies have reported that some COVID-19 convalescent individuals who met the criteria of hospital discharge had positive RT-PCR test results again [10]. The IgG and neutralizing antibody (NAb) titers of a high portion of COVID-19 convalescents have dropped swiftly within 2–3 months [11], indicating a short protection period after recovery from SARS-CoV-2 infection. Furthermore, T cell responses could provide a long protection time for patients [12]. It is critical to surveil the dynamic changes in NAbs, as well as the cellular immune response induced by the COVID-19 vaccine.

The receptor binding domain (RBD), located at the spike (S) gene, is responsible for binding to the angiotensin converting enzyme 2 (ACE2) receptor of host cell [13]. Most of the NAbs target RBD [14,15], and partial antigen epitopes in other domains of the S protein may even cause the antibody-dependent effects (ADEs) [16]. Some vaccines, such as MERS and SARS subunit vaccines, were developed based on the RBD protein [17,18]. The RBD protein seems to be an effective and safe antigen candidate. However, the immunogenicity of RBD monomers is not strong enough [19]. RBD-sc-dimer with a tandem repeat single-chain has proved to induce an effective protection against COVID-19 in both pre-clinical experiments and clinical trials [19]. Fc-fusion protein, which can form RBD-Fc dimers and effectively prolong the metabolism time in vivo [20], might increase the immunogenicity of RBD monomers. A recombinant vaccine based on the RBD-Fc fusion protein could induce good humoral immune response in both nonhuman primates and mice [21].

In this study, compared with the RBD-Fc fusion protein study previously [21], we first used HEK293T cells to express the RBD protein linked with the modified immunoglobulin Fc fragment to produce a recombinant protein vaccine in the Fc fusion protein form. To reduce the immune rejection in mice and estimate the efficacy of this fusion protein vaccine, RBD fused with mouse Fc protein was also used in this study. We measured the longitudinal dynamics of the IgG and neutralizing antibody responses for three months. Both humoral and cellular immune responses were detected in recombinant RBD subunit vaccine-immunized mice. Here, we demonstrated an effective subunit vaccine based on the RBD-Fc fusion protein.

## 2. Materials and Methods

### 2.1. Ethics Statements

The animal experiments were approved by Animal Ethical and Welfare Committee of Zhejiang Chinese Medical University (IACUC-20200323-07). All the experiments related to live SARS-CoV-2 viruses were carried out in biosafety level 3 (BSL-3) laboratory in Zhejiang Provincial Center for Disease Control and Prevention.

### 2.2. Virus and Cell Lines

The SARS-CoV-2 clinical strain 12# used in this study was purified from the sputum of a male COVID-19 patient in Wenzhou as mentioned previously [22]. The 50% tissue culture infective dose (TCID_50_) was calculated using Reed and Muench methods [23]. Human embryonic kidney cells (HEK293T) and Vero E6 cells were purchased from the National Collection of Authenticated Cell Cultures. Cells were maintained in Dulbecco’s Modified Eagle’s Medium (Gibco, Waltham, MA, USA) or Minimal Essential Medium (Gibco, Waltham, MA, USA), supplemented with 10% fetal bovine serum (FBS, Gibco, Waltham, MA, USA), 100 U/mL penicillin (Solarbio, Beijing, China), 100 mg/mL streptomycin (Solarbio, Beijing, China) at 37 °C with 5% CO_2_.

### 2.3. Gene Cloning, Expression and Purification of Recombinant Proteins

The coding sequence of the RBD region (S protein 330-583, NCBI Reference Sequence: NC_045512.2) was codon-optimized for mammalian cell expression and synthesized (Sangon, Shanghai, China). For the construction, the signal peptide sequence was added to the N-terminus of the RBD sequence and the Fc fragment of mouse IgG1 or human IgG1 (referred to as hFc or mFc) followed by G_4_S linker added to the C-terminus of the RBD sequence (Supplementary Tables S1 and S2). In order to reduce the potential adverse effects such as antibody-dependent cell-mediated cytotoxicity (ADCC) elicited by human IgG1, the Fc fragment was modified by N297A and K322A as previously described [24]. Then, the construct was inserted into the pcDNA3.1 expression vector via the Hind-III and EcoR-I restriction sites. HEK293T cells were cultured in T75 flasks (Thermo Fisher, Waltham, MA, USA), and the recombinant plasmid was transfected into HEK293T cells through PEI (Polysciences, Niles, Illinois, USA). After 3~5 days, the supernatant was collected and first purified by Protein G or Protein A affinity chromatography (GE Healthcare, Shanghai, China), and further purified by SEC (Superdex^TM^ 75 Increase 10/300 GL, GE healthcare, Waltham, MA, USA) for the RBD recombinant protein. The S1-hFc protein (40591-V02H) was obtained from the Sino Biological Company (Beijing, China).

### 2.4. Sodium Dodecyl Sulfate Polyacrylamide Gel Electrophoresis (SDS-PAGE)

The 12% protein gel was used to characterize the molecular weight and purity of RBD recombinant protein. In short, purified RBD recombinant protein (10 μg) plus 4 μL loading buffer containing 1% β-mercaptoethanol (β-ME) was boiled for 6~7 min, and then analyzed by SDS-PAGE. Another 10 μg non-boiled RBD recombinant plus 4 μL loading buffer without β-ME was analyzed by SDS-PAGE directly. The parameters of protein electrophoresis were as follows: concentrated gel 90 V, 25 min; separation gel 130 V, 1.5 h. The protein was dyed in Coomassie brilliant blue for 1–2 h at room temperature. After dyeing, the gel was placed in the decolorizing solution until the strip was clearly visible.

### 2.5. Flow Cytometry Analysis for Vero E6 Cell Binding Ability

Vero E6 cells were washed with pre-cooled PBS buffer (pH~7.4) and incubated with 10 μg/mL purified RBD-hFc or RBD-mFc recombinant protein for 30 min on ice. After a third wash, the cells were incubated with FITC-labeled goat anti-human IgG or FITC-labeled goat anti-mouse IgG, diluted 1000-fold in 200 μL. Finally, the mean fluorescence intensity (MFI) was measured on an ACEA NovoCyteTM flow cytometer.

### 2.6. Mouse Experiments

**Figure 1:**
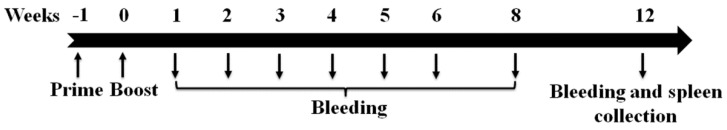
Timeline of the immunization and blood collection. BALB/c mice were immunized with high and low doses of RBD-hFc, RBD-mFc and S1-hFc proteins mixed with 0.5 mg/mL aluminum hydroxide adjuvant each and boosted 1 week later. Blood were taken at 1, 2, 3, 4, 5, 6, 8 and 12 weeks after the second vaccination. Spleens were collected after euthanizing.

Eight-week-old, female BALB/c mice were randomly divided into seven groups, and each group contained four mice. High (8 μg) and low (2 μg) doses of RBD-hFc, RBD-mFc and S1-hFc proteins mixed with 0.5 mg/mL aluminum hydroxide adjuvant each were used to immunize mice intramuscularly. The immune program was vaccinated at day 0 and boosted at day 7. Blood were taken as the schedule below, and the spleens were collected after euthanizing (Figure 1). Aluminum hydroxide at a concentration of 0.5 mg/mL in PBS was used as the control.

### 2.7. Enzyme-Linked Immunosorbent Assay (ELISA)

In short, microwells of ELISA plates (Corning, NY, USA) were coated with 50 ng RBD protein each (Genscript, Nanjing, China) at 4 °C overnight. Plates were blocked with 10% FBS in PBS for 1 h at 37 °C, and then incubated with 2-fold serially diluted serum samples for another hour. After washing three times, plates were incubated with rabbit anti-mouse IgG-HRP antibody at a dilution of 1:20,000 (Abcam, Cambridge, UK) at 37 °C for 1 h. Tetramethylbenzidine (Solarbio, Beijing, China) and hydrogen peroxide were used for color development, and the reaction was stopped with 2 M H_2_SO_4_. The absorbance was measured at 450 nm, and an optical density at 450 nm (OD_450_) value greater than 2.1-fold of the background value was regarded as positive [25].

### 2.8. Neutralization Assay

The plaque reduction neutralization test (PRNT) was conducted as the neutralization assay. Briefly, sera from immunized mice were inactivated at 56 °C for 0.5 h. Serially 2-fold diluted sera were mixed with the same volume of 12# SARS-CoV-2 (100 TCID_50_) virus culture and incubated for 1 h at 37 °C. Then, the virus–serum mixture was transferred to pre-plated Vero E6 cells in 6-well plates. After incubation at 37 °C for another 1 h, the mixture was discarded, and the cells were coated with a 0.6% agarose gel in the virus culture medium. Two days later, the second agarose layer containing 0.1% neutral red was added. Plaque numbers were counted one day later, and the neutralization titers were calculated as the reciprocal of serum dilutions leading to 50% plaque reductions (PRNT_50_).

### 2.9. Enzyme-Linked Immunospot Assay (ELISPOT)

Twelve weeks after the boost immunization, all mice from the seven groups were euthanized. Spleens were collected and teased apart into single splenocyte suspensions by pressing through a 3 mL syringe. Splenocytes were cultured in an IFN-γ antibody pro-coated ELISPOT plate (BD Biosciences, NJ, USA) at a density of 1 × 10^6^ per well and stimulated with or without SARS-CoV-2 RBD (2 μg/well). Following incubation at 37 °C in 5% CO_2_ for 16 h, splenocytes producing IFN-γ were measured using mouse enzyme-linked immunospot (ELISPOT) kits (BD Biosciences, San Jose, NJ, USA) according to the manufacturer’s instructions. Spot-forming cells (SFCs) were imaged by a ChemiDoc XRS+ imaging system (Bio-Rad, Hercules, CA, USA), and the related data were statistically analyzed by Quantity One software.

### 2.10. IL-4 and IL-10 Detection

Splenocytes were prepared as described in the ELISPOT section and seeded in a 96-well plate at a density of 1 × 10^6^ per well. After stimulation with or without SARS-CoV-2 RBD (2 μg/well) at 37 °C in 5% CO_2_ for 16 h, the supernatants of each well were collected and the levels of secreted IL-4 and IL-10 were detected by ELISA (BD Biosciences, San Jose, NJ, USA) kits.

### 2.11. Statistical Analysis

All data were analyzed with GraphPad Prism 8.0. The Student’s *t*-test was performed with *p* < 0.05 between two groups considered as statistically significant.

## 3. Results

### 3.1. Characterization of the SARS-CoV-2 RBD Recombinant Proteins

**Figure 2:**
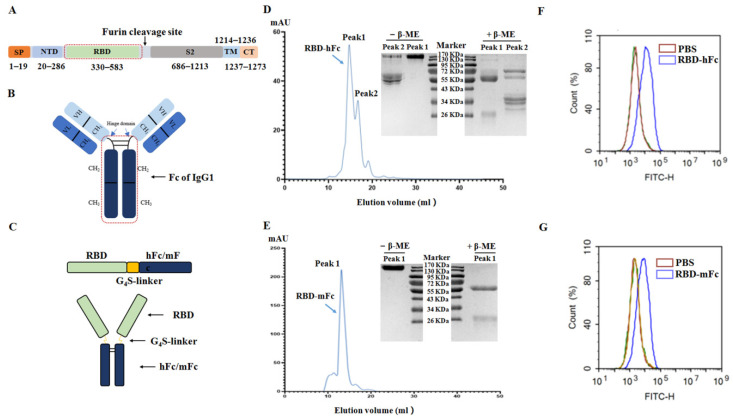
Construction and characterization of SARS-CoV-2 RBD recombinant proteins. (**A**) A schematic view of the SARS-CoV-2 S protein. RBD ranges from 330 to 583 aa of the SARS-CoV-2 S protein. SP, signal peptide; NTD, N-terminal domain; TM, transmembrane; CT, C-terminal domain. (**B**) Structure diagram of IgG1. Fc contains the hinge domain, CH_2_ and CH_3_. (**C**) A schematic view of the SARS-CoV-2 recombinant protein. Residues 330–583 aa of the SARS-CoV-2 S protein were fused with a modified Fc fragment of human IgG1 or mouse IgG1 (hFc or mFc) via a G_4_S (Gly-Gly-Gly-Gly-Ser) flexible linker and engineered in a mammalian cell expression system. The purification results of RBD-hFc recombinant protein (**D**) and RBD-mFc recombinant protein (**E**) after purified by the protein A/G chromatographic column and a Superdex 200 increase column. Each elution peak was analyzed by SDS-PAGE in the presence of β-mercaptoethanol (β-ME) or not. The elution peaks of both recombinant proteins were noted with blue arrows. The cell binding ability of the RBD-hFc recombination protein (**F**) and RBD-mFc recombinant protein (**G**) were measured by flow cytometry using the Vero E6 cell line.

The RBD, spanning from residues 330–583 of the spike protein of the SARS-CoV-2 (Figure 2A), was fused with a modified Fc fragment of mouse IgG1 or human IgG1 (Figure 2B) to form RBD-hFc or RBD-mFc proteins (Figure 2C). These two types of SARS-CoV-2 RBD recombinant protein were successfully harvested from culture supernatant of transfected HEK293T cells, and then purified using a protein A/G chromatographic column and a Superdex 200 increase column (Figure 2D,E). The reduced and non-reduced of RBD-hFc and RBD-mFc recombinant proteins were analyzed by SDS-PAGE (right of Figure 2D,E). To further determine the binding ability of SARS-CoV-2 RBD recombinant protein to ACE2 receptor, Vero E6 cells containing the ACE2 receptor were incubated with the RBD recombinant protein and analyzed by flow cytometry. Compared with the control group, the fluorescence of the incubated RBD recombinant protein group was significantly shifted (Figure 2F,G), indicating the binding ability of RBD recombinant proteins to the ACE2 receptor. The results showed that the RBD-hFc and RBD-mFc recombinant proteins with their biological function remaining were obtained successfully.

### 3.2. The Immunogenicity of Recombinant RBD and S1 Subunit Vaccines

**Figure 3:**
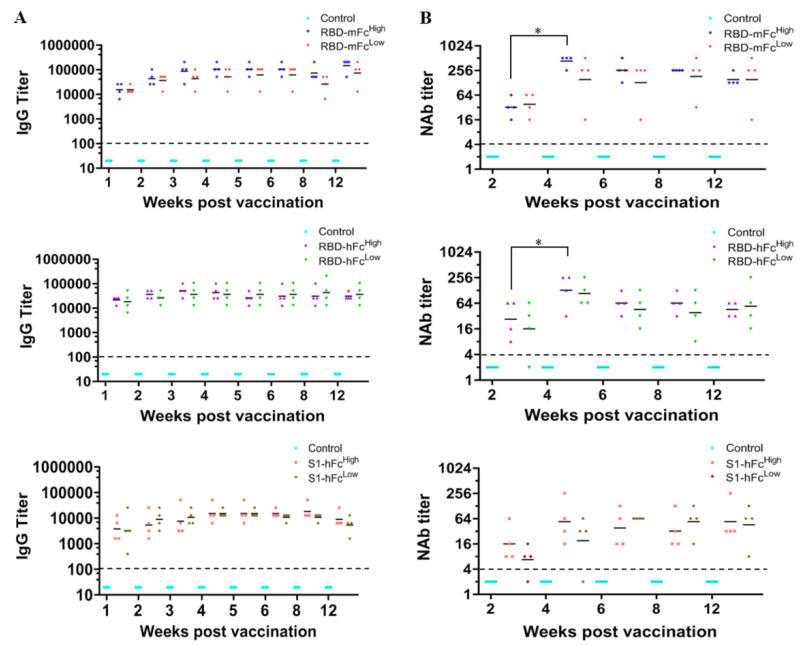
The immunogenicity of recombinant RBD and S1 subunit vaccines. BALB/c mice were immunized with high (8 μg) or low (2 μg) doses of RBD-mFc, RBD-hFc and S1-hFc fusion vaccines. Aluminum hydroxide in PBS was used as the negative control. Following a two-dose immunization program, serum samples were collected to assess the humoral immunity. (**A**) ELISA was performed to measure IgG antibody titers. (**B**) The plaque reduction neutralization test (PRNT) was conducted as the neutralization assay, and the neutralization antibody (NAb) titers were calculated as the reciprocal of serum dilutions leading to 50% plaque reductions (PRNT_50_). The dotted lines meant the detection limit of this assay. * Significant difference between the two groups (*p* < 0.05).

To measure the immunogenicity of RBD-hFc and RBD-mFc subunit vaccines, BALB/c mice were immunized with high and low doses of fusion proteins in a two-dose immunization program. The immunogenicity of S1-hFc fusion protein was also detected. As shown in Figure 3A, one week after the booster vaccination, the IgG seroconversion rate was 100% in all groups. The IgG antibody titers rose and reached a peak at 3–4 weeks post vaccination. The highest geometric mean titers (GMTs) of IgG antibody in the RBD-mFc^High^, RBD-mFc^Low^, RBD-hFc^High^, RBD-hFc^Low^, S1-hFc^High^ and S1-hFc^Low^ groups were 144,816, 72,408, 51,200, 36,204, 18,102, and 15,222, respectively. Over time, IgG antibody titers decreased slightly but still sustained at a high level at 12 weeks post vaccination. The IgG antibody levels in both the RBD-mFc and RBD-hFc groups were higher than those in the S1-hFc group.

As shown in Figure 3B, the neutralizing antibody titer could be detected at two weeks post vaccination in all groups except one mouse in the S1-hFc^Low^ group and another one in the RBD- hFc^Low^ group. The neutralizing antibody titers peaked at four weeks post vaccination, similar to the IgG antibody titers, with the GMTs of 431, 152, 128, 108 and 54 in the RBD-mFc^High^, RBD-mFc^Low^, RBD-hFc^High^, RBD-hFc^Low^ and S1-hFc^High^ groups, respectively. The GMTs of the RBD-mFc^High^ and RBD-hFc^High^ groups rose significantly at four weeks post-vaccination compared to two weeks. The highest GMT in the S1-hFc^Low^ group was 64, a six-week interval after vaccination. Then, the neutralizing antibody titer reduced. At 12 weeks post vaccination, the GMTs in the RBD-mFc^High^, RBD-hFc^High^, RBD-hFc^Low^ and S1-hFc^Low^ groups were 35.4%, 35.4%, 50% and 70.3% of the highest GMTs, respectively. However, in the RBD-mFc^Low^ group, the neutralizing antibody level was nearly the same as the peak level. The results showed that the subunit vaccine based on RBD fusion proteins had good immunogenicity in mice, and the immunogenicity of RBD fusion proteins were better than the S1 fusion protein.

### 3.3. Cellular Immune Response of Recombinant RBD and S1 Subunit Vaccines

**Figure 4:**
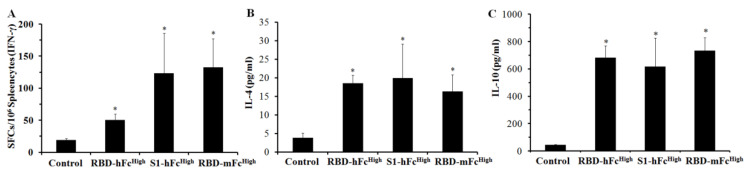
Cellular immune response of recombinant RBD and S1 subunit vaccines. Twelve weeks after the boost immunization, the splenocytes isolated from RBD-hFc, RBD-mFc and S1-hFc fusion protein-immunized mice were stimulated with the RBD protein for 16 h. (**A**) ELISPOT was conducted to evaluate the concentration of IFN-γ secreted by splenocytes. ELISA was performed to quantify the release of IL-4 (**B**) and IL-10 (**C**) in the culture medium of splenocytes. * Significant difference compared with the control group (*p* < 0.05).

To evaluate the cellular immune response of the recombinant RBD and S1 subunit vaccines, splenocytes were separated from all immunized mice and stimulated with the RBD protein overnight. Using ELISPOT detection, the secretion level of IFN-γ was significantly higher than that of the control, with the RBD-mFc^High^ group having the highest secretion level among the four groups (Figure 4A). IL-4 and IL-10 excreted by the RBD-stimulated splenocytes in the culture medium were measured by ELISA. As shown in Figure 4B,C, the concentrations of IL-4 and IL-10 in the RBD-mFc^High^, RBD-hFc^High^ and S1-hFc^High^ groups were also significantly higher than those in the control groups. The IL-4 concentrations of these three groups were 16.3, 18.6 and 19.9 pg/mL, while the IL-10 concentrations were 733.7, 683.3 and 617.4 pg/mL, respectively. These results indicated that the subunit vaccine based on RBD fusion proteins could trigger a cellular immune response in mice.

## 4. Discussion

The COVID-19 pandemic, caused by the SARS-CoV-2, is a novel respiratory disease and has posed a serious threat to global public health. Here, we focused on a subunit vaccine based on an RBD-Fc fusion protein and first explored the longitudinal dynamics of the IgG and neutralizing antibody responses. We proved the feasibility of our recombinant RBD-Fc subunit vaccine candidate, which could induce IgG and NAb responses effectively and last for at least three months in mice. Moreover, it is encouraging to observe that it can also trigger the cellular immune response.

The RBD domain of the S protein engages with the host ACE2 receptor and accomplishes the first important step during infection [13]. Moreover, the RBD domain is the most concentrated area of epitopes (Supplementary Figure S1) and has been regarded as an attractive immunogen [26,27,28]. However, compared with the RBD-dimer, the RBD monomer has poor immunogenicity and triggers low levels of IgG and neutralization titers [19]. In this study, we added the Fc fragment to the C-terminus of the RBD to form a RBD-Fc fusion protein, which could not only increase the molecular weight by 2-fold to be similar as the RBD-dimer, but also stimulate B-cells through the Fc receptor [20]. The immunogenicity of the RBD fusion protein group was good, and much better than the RBD monomer group (Supplementary Figure S2). S1 protein, one of the two subunit of S protein, contains the whole RBD and several neutralization epitopes outside the RBD [29]. However, the S1 protein induced a lower level of neutralization titer than the RBD protein [30]. Similar results were also found in our study. Compared with the RBD-hFc or RBD-mFc group, vaccine based on the S1-hFc protein induced a lower level of IgG and neutralization titers in BALB/c mice. It seemed that RBD might be a better candidate than S1 for SARS-CoV-2 vaccine design. What is more, S protein, containing the S1 domain, seems a good vaccine candidate like RBD. Another COVID-19 vaccine candidate NVX-CoV2373 (Novavax), based on the full-length of S protein, could trigger a robust immune response in human beings like the dimeric RBD-based protein subunit vaccine (ZF2001) [9,31].

To reduce the potential adverse effect of Fc protein such as ADCC originating from human IgG1, we designed the modified Fc protein by N297A and K322A mutations as previously described, different from the native Fc protein used in another RBD-Fc fusion protein study [21,24]. To reduce the reject reaction of human Fc in mice, we also measured the mouse Fc-fused RBD. Levels of IgG and neutralization titers in the RBD-mFc group were a little higher than the RBD-hFc group, indicating a good prospect of RBD-hFc vaccine in human beings. Moreover, Fc effectively promotes recombinant protein generation via a mammalian expression system (HEK293T) that retains complex glycosylation modification, which has an advantage over yeast or prokaryotic expression systems.

To further evaluate the immunogenicity of recombinant RBD vaccine candidates, we analyzed the dynamic changes in specific IgG and NAb titers. It is impressive that the IgG titer in the RBD-hFc and RBD-mFc groups maintained a high level for three months. Although the NAb titers in the RBD-hFc^High^, RBD-hFc^Low^, RBD-mFc^High^ and S1-hFc^Low^ groups decreased significantly at 12 weeks post vaccination, the GMTs of these four groups were still all above 32. A similar phenomenon could be found in convalescent COVID-19 patients, with a mean NAb titer reduction of 34.8% [32]. However, it was interesting to find that the neutralizing antibody levels at 12 weeks post vaccination in the RBD-mFc^Low^ group were nearly the same as the peak levels. It implied that a proper immune dose was important for the long-term immunogenicity of recombinant RBD vaccine candidates.

The cellular immune response is critical for virus control and clearance in acute infection [30,33]. Through the ELISPOT and ELISA detection, we found that splenocytes isolated from the vaccine-immunized BALB/C mice could secrete higher levels of IFN-γ, IL-4 and IL-10 when stimulated with the RBD protein, indicating a robust cellular immune response. However, IL-2, another cytokine, increased not significantly in our study (Supplementary Figure S3). The result was different from another study based on the RBD-Fc Vacc-vaccinated Macaca fascicularis macaques, with no significant cellular immune response detected [21]. Different animal model might be the reason to explain the difference. IFN-γ is mainly secreted by T helper 1 (Th1) cells, which could be responsible for cell-mediated immunity, while IL-4 and IL-10 are mainly secreted by T helper 2 (Th2) cells, which can induce significant antibody production [34]. Combined with the high NAb level in the immunized mice, the recombinant RBD vaccine could induce both humoral and cellular immune responses in mice. Coordination between humoral and cellular immune responses could limit the COVID-19 disease severity better than partial responses [35], indicating a promising vaccine of the RBD-Fc fusion protein. Additionally, the safety of the recombinant RBD vaccine was evaluated by histological examination. No obvious lesions were detected in the lung and kidney sections of immunized mice (Supplementary Figure S4). However, owing to the facility limitations, we did not detect the protective efficacy of this vaccine. It should be unveiled in future studies.

Last, SARS-CoV-2 is an RNA virus that has a high mutation rate. With the evolution of the SARS-CoV-2 virus, some variants such as alpha variant (B.1.1.7) and beta variant (B.1.351), have increased the binding affinity to the ACE2 receptor and resistant to the neutralizing antibodies [36,37]. Among the variants, the delta variant (B.1.617.2), with a higher potential rate of transmission than other variants [38], has been reported to have spread to over 100 countries, leading to it being more difficult to control the COVID-19 pandemic. The generation of the recombinant RBD-Fc fusion vaccine is simple and fast, which could be easily prepared for different SARS-CoV-2 variants. Given the similarity of SARS-CoV-2, SARS [39], and MERS, we further speculated that the recombinant RBD-Fc vaccine may also be suitable for other β-coronaviruses. Moreover, the function of the Fc protein can be applied to combine two different RBD regions derived from SARS-CoV-2 mutations or other β-coronaviruses that may be a universal vaccine for β-coronaviruses.

In summary, our study constructed an RBD-Fc fusion protein and demonstrated it as a promising SARS-CoV-2 vaccine candidate. The RBD-Fc vaccine candidate could induce both humoral and cellular immune responses, with high levels of IgG and neutralizing antibody lasting for at least three months. This recombinant vaccine candidate, with its simple, rapid and economical preparation, might be another optional strategy to control COVID-19 spread.

## Supplementary Materials

The following are available online at https://www.mdpi.com/article/10.3390/v13101936/s1, Figure S1: The epitopes with high immunogenicity on the RBD, Figure S2: The immunogenicity of RBD vaccine, Figure S3: The expression of IL-2 in the recombinant RBD and S1 subunit vaccines-immunized mice, Figure S4: Representative images of haematoxylin and eosin stained lungs and kidneys isolated from the RBD-hFc, RBD-mFc and S1-hFc fusion vaccines immunized mice at 12 weeks post vaccination. Table S1: The gene sequence of RBD-hFc, Table S2: The gene sequence of RBD-mFc.
