# Supplementary material for: Development of a Recombinant RBD Subunit Vaccine for SARS-CoV-2
Source: Viruses. 2021 Sep 26;13(10):1936. doi: 10.3390/v13101936 (PMC8541238; doi:10.3390/v13101936)
Supplement: Supplementary file 1 [file viruses-13-01936-s001.zip › viruses-1369581-supplementary.pdf]

Development of a recombinant RBD subunit vaccine for SARS-CoV-2

Yi-Sheng Sun[1,+], Jing-Jing Zhou[2,3+], Han-Ping Zhu[1], Fang Xu[1], Wen-Bin Zhao[2,3], Hang-Jing Lu[1], Zhen Wang[1], Shu-Qing Chen[2], Ping-Ping Yao[1*], Jian-Min Jiang[1*], Zhan Zhou[2,3,4*]

1 Key Lab of Vaccine, Prevention and Control of Infectious Disease of Zhejiang Province, Zhejiang Provincial Center for Disease Control and Prevention, Hangzhou 310015, China

2 Institute of Drug Metabolism and Pharmaceutical Analysis and Zhejiang Provincial Key Laboratory of Anti-Cancer Drug Research, College of Pharmaceutical Sciences, Zhejiang University, Hangzhou 310058, China

3 Innovation Institute for Artificial Intelligence in Medicine, Zhejiang University, Hangzhou 310018, China

4 Alibaba-Zhejiang University Joint Research Center of Future Digital Healthcare, Hangzhou 311121, China

* Corresponding authors: Ping-Ping Yao, Email: ppyao@cdc.zj.cn; Jian-Min Jiang, jmjiang@cdc.zj.cn; Zhan Zhou, zhanzhou@zju.edu.cn

+ These authors made equal contributions to this work.

Table S1. The gene sequence of RBD-hFc.

AAGCTTACCACCATGGATATGAGGGTGCCTGCCCAGCTGCTGGGACTGCTCCTGCTGTGGTT
TCCCGGCGCCAGATGCCCTAACATTACCAACCTCTGCCCATTTGGAGAGGTGTTTAACGCCA
CCCGGTTCGCCAGCGTGTACGCCTGGAACCGGAAGAGGATCAGCAACTGCGTGGCCGACTA
CAGCGTGCTGTACAACAGCGCCTCCTTCAGCACCTTCAAGTGCTACGGGGTGAGCCCCACA
AAGCTGAACGATCTGTGCTTCACCAACGTATACGCCGATAGCTTCGTGATCCGGGGGGATG
AGGTGAGGCAGATCGCCCCCGGCCAGACAGGCAAGATCGCCGATTACAACTACAAGCTGCC
CGATGACTTCACCGGCTGCGTGATCGCCTGGAACAGCAACAACCTGGACTCCAAGGTGGGC
GGCAACTACAACTACCTGTACCGCCTGTTCAGGAAGTCCAACCTGAAGCCTTTTGAGAGGG
ATATCAGCACAGAGATCTACCAGGCCGGCTCCACACCCTGCAACGGCGTGGAGGGGTTCAA
CTGCTACTTCCCCCTGCAGAGCTATGGCTTCCAGCCCACAAACGGGGTGGGGTACCAGCCCT
ACAGGGTGGTGGTGCTGAGCTTCGAGCTGCTGCACGCCCCCGCCACAGTGTGCGGGCCCAA
GAAGTCCACCAACCTGGTGAAAAACAAGTGCGTGAACTTCAACTTCAACGGGCTGACAGGG
ACCGGCGTGCTGACAGAGAGCAACAAGAAGTTCCTGCCCTTCCAGCAGTTCGGGCGGGATA
TCGCCGACACCACAGACGCCGTGAGGGACCCCCAGACACTGGAGGGAGGAGGCGGCAGCC
CCAAATCTTGTGACAAAACTCACACATGCCCACCGTGCCCAGCACCTGAAGCCGCTGGGGG
ACCGTCAGTCTTCCTCTTCCCCCCAAAACCCAAGGACACCCTCATGATCTCCCGGACCCCTG
AGGTCACATGCGTGGTGGTGGACGTGAGCCACGAAGACCCTGAGGTCAAGTTCAACTGGTA
CGTGGACGGCGTGGAGGTGCATAATGCCAAGACAAAGCCGCGGGAGGAGCAGTACGCCAG
CACGTACCGTGTGGTCAGCGTCCTCACCGTCCTGCACCAGGACTGGCTGAATGGCAAGGAG
TACAAGTGCGCCGTCTCCAACAAAGCCCTCGGAGCCCCCATCGAGAAAACCATCTCCAAAG
CCAAAGGGCAGCCCCGAGAACCACAGGTGTACACCCTGCCCCCATCCCGGGATGAGCTGAC
CAAGAACCAGGTCAGCCTGACCTGCCTGGTCAAAGGCTTCTATCCCAGCGACATCGCCGTG
GAGTGGGAGAGCAATGGGCAGCCGGAGAACAACTACAAGACCACGCCTCCCGTGCTGGAC
TCCGACGGCTCCTTCTTCCTCTACAGCAAGCTCACCGTGGACAAGAGCAGGTGGCAGCAGG
GGAACGTCTTCTCATGCTCCGTGATGCATGAGGCTCTGCACAACCACTACACGCAGAAGAG
CCTCTCCCTGTCTCCGGGTAAATGAGAATTC

The restriction site (Hind-III and EcoR-I) was marked in red font; The Kozak sequence was marked in brown font; The signal sequence was marked in orange font; The codon-optimized RBD sequence was marked in black font; The flexible linker was marked in purple font; The hFc sequence was marked in blue font; The amino acid of Fc mutation is highlighted with yellow.

Table S2. The gene sequence of RBD-mFc.

AAGCTTACCACCATGGATATGAGGGTGCCTGCCCAGCTGCTGGGACTGCTCCTGCTGTGGTT
TCCCGGCGCCAGATGCCCTAACATTACCAACCTCTGCCCATTTGGAGAGGTGTTTAACGCCA
CCCGGTTCGCCAGCGTGTACGCCTGGAACCGGAAGAGGATCAGCAACTGCGTGGCCGACTA
CAGCGTGCTGTACAACAGCGCCTCCTTCAGCACCTTCAAGTGCTACGGGGTGAGCCCCACA
AAGCTGAACGATCTGTGCTTCACCAACGTATACGCCGATAGCTTCGTGATCCGGGGGGATG
AGGTGAGGCAGATCGCCCCCGGCCAGACAGGCAAGATCGCCGATTACAACTACAAGCTGCC
CGATGACTTCACCGGCTGCGTGATCGCCTGGAACAGCAACAACCTGGACTCCAAGGTGGGC
GGCAACTACAACTACCTGTACCGCCTGTTCAGGAAGTCCAACCTGAAGCCTTTTGAGAGGG
ATATCAGCACAGAGATCTACCAGGCCGGCTCCACACCCTGCAACGGCGTGGAGGGGTTCAA
CTGCTACTTCCCCCTGCAGAGCTATGGCTTCCAGCCCACAAACGGGGTGGGGTACCAGCCCT
ACAGGGTGGTGGTGCTGAGCTTCGAGCTGCTGCACGCCCCCGCCACAGTGTGCGGGCCCAA
GAAGTCCACCAACCTGGTGAAAAACAAGTGCGTGAACTTCAACTTCAACGGGCTGACAGGG
ACCGGCGTGCTGACAGAGAGCAACAAGAAGTTCCTGCCCTTCCAGCAGTTCGGGCGGGATA
TCGCCGACACCACAGACGCCGTGAGGGACCCCCAGACACTGGAGGGAGGAGGCGGCAGCG
TTAGATCTGGTTGTAAGCCTTGCATATGTACAGTCCCAGAAGTATCATCTGTCTTCATCTTCC
CCCCAAAGCCCAAGGATGTGCTCACCATTACTCTGACTCCTAAGGTCACGTGTGTTGTGGTA
GACATCAGCAAGGATGATCCCGAGGTCCAGTTCAGCTGGTTTGTAGATGATGTGGAGGTGC
ACACAGCTCAGACGCAACCCCGGGAGGAGCAGTTCAACAGCACTTTCCGCTCAGTCAGTGA
ACTTCCCATCATGCACCAGGACTGGCTCAATGGCAAGGAGTTCAAATGCAGGGTCAACAGT
GCAGCTTTCCCTGCCCCCATCGAGAAAACCATCTCCAAAACCAAAGGCAGACCGAAGGCTC
CACAGGTGTACACCATTCCACCTCCCAAGGAGCAGATGGCCAAGGATAAAGTCAGTCTGAC
CTGCATGATAACAGACTTCTTCCCTGAAGACATTACTGTGGAGTGGCAGTGGAATGGGCAG
CCAGCGGAGAACTACAAGAACACTCAGCCCATCATGGACACAGATGGCTCTTACTTCGTCTA
CAGCAAGCTCAATGTGCAGAAGAGCAACTGGGAGGCAGGAAATACTTTCACCTGCTCTGTG
TTACATGAGGGCCTGCACAACCACCATACTGAGAAGAGCCTCTCCCACTCTCCTGGTAAATG
AGAATTC

The restriction site (Hind-III and EcoR-I) was marked in red font; The Kozak sequence was marked in brown font; The signal sequence was marked in orange font; The codon-optimized RBD sequence was marked in black font; The flexible linker was marked in purple font; The mFc sequence was marked in blue font.

Supplementary Figure 1

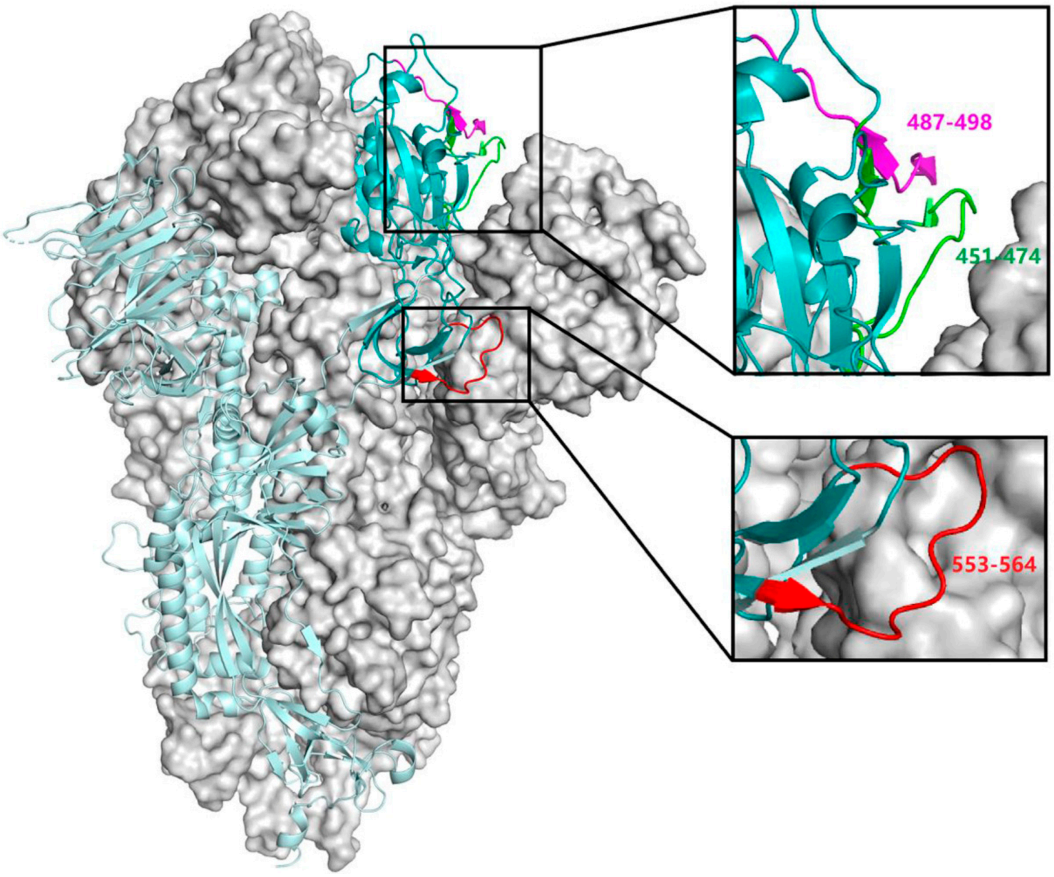

Figure S1. The epitopes with high immunogenicity on the RBD. Grey, S protein; Green strip, RBD; Purple, green and red strips, epitopes with high immunogenicity on the RBD; Cyan strip, other domain except RBD.

Supplementary Figure 2

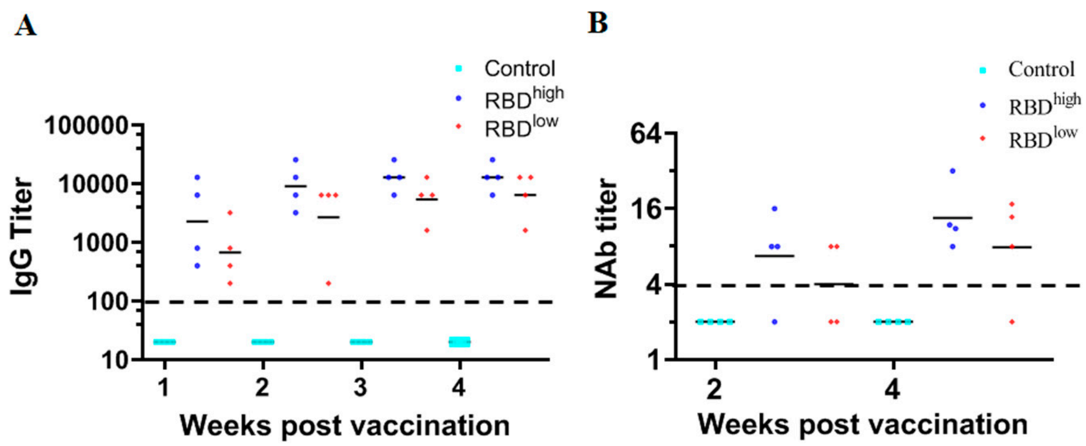

Figure S2. The immunogenicity of RBD vaccine. High (8 μg) or low (2 μg) doses of RBD proteins (Genscript, Nanjing, China) mixed with 0.5 mg/ml aluminum hydroxide adjuvant were used as RBD vaccines. Eight-week-old female BALB/c mice were immunized with high or low doses of RBD vaccines, and aluminum hydroxide in PBS was used as the negative control. The immune program was vaccinated at day 0 and boosted at day 7. One to four weeks after booster vaccination, serum samples were collected to assess humoral immunity. (A) ELISA was performed to measure the IgG antibody titers. (B) The plaque reduction neutralization test (PRNT) was conducted as the neutralization assay, and the neutralization antibody (NAb) titers were calculated as the reciprocal of serum dilutions leading to 50% plaque reductions ($PRNT_{50}$). The dotted lines meant the detection limit of this assay.

Supplementary Figure 3

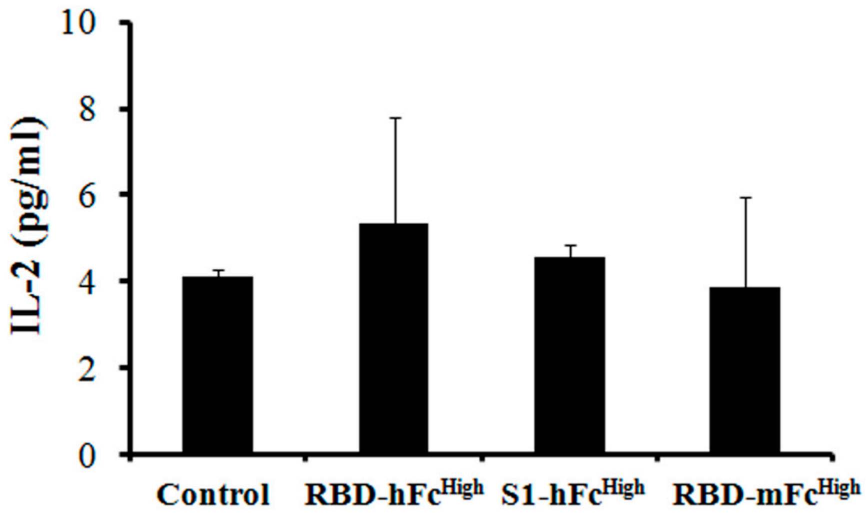

Figure S3. The expression of IL-2 in the recombinant RBD and S1 subunit vaccines-immunized mice. Twelve weeks after the boost immunization, the splenocytes isolated from RBD-hFc, RBD-mFc and S1-hFc fusion protein-immunized mice were stimulated with the RBD protein for 16 hours. ELISA was performed to measure the release of IL-2 in the culture medium of splenocytes.

Supplementary Figure 4

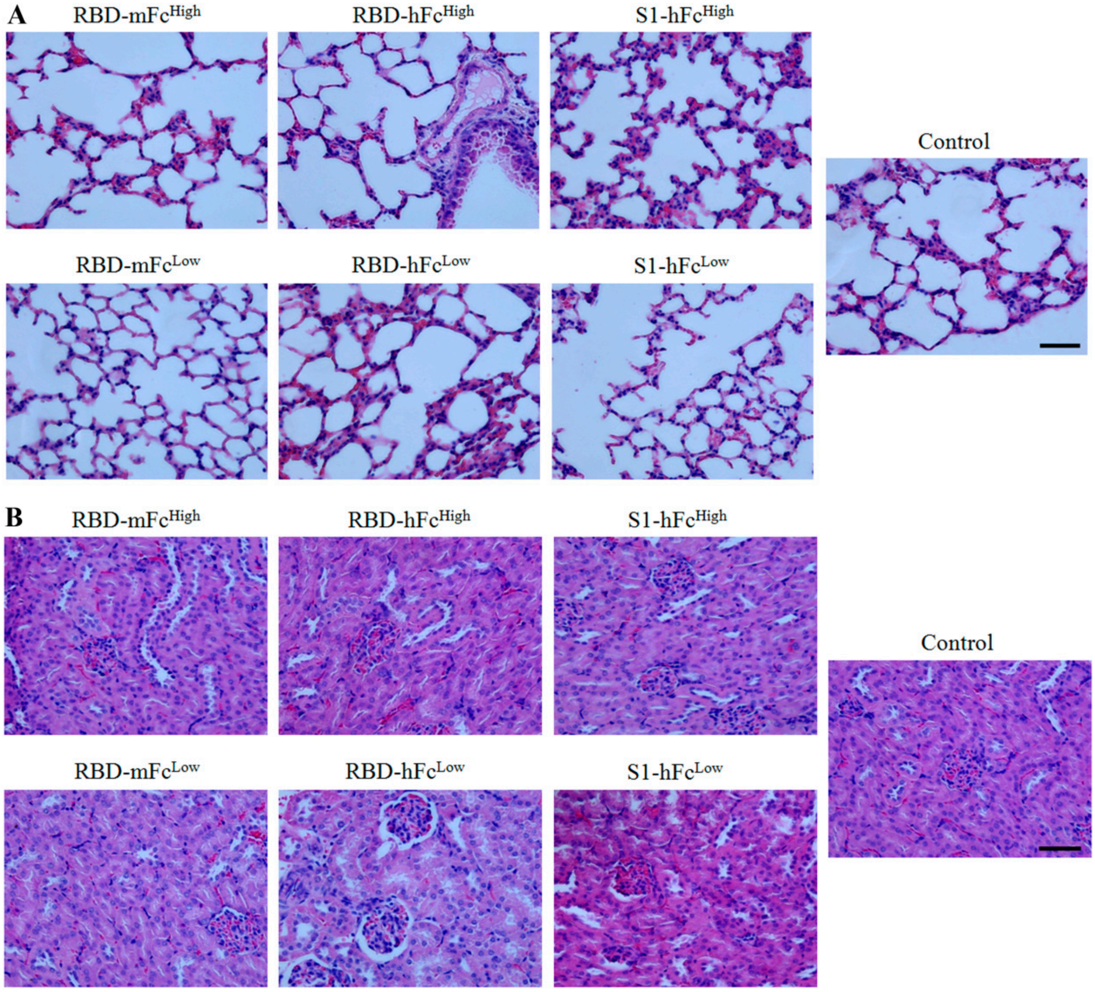

Figure S4. Representative images of haematoxylin and eosin stained lungs and kidneys isolated from the RBD-hFc, RBD-mFc and S1-hFc fusion vaccines immunized mice at 12 weeks post vaccination. Mice immunized with aluminum hydroxide in PBS were used as the negative control. Bar: 50 μm. (A): Lung sections. (B): Kidney sections.
